# Genome Sequence of the Mosquitocidal *Bacillus thuringiensis* Strain BR58, a Biopesticide Product Effective against the Coffee Berry Borer (*Hypothenemus hampei*)

**DOI:** 10.1128/genomeA.01232-15

**Published:** 2015-12-10

**Authors:** Janaina Zorzetti, Ana P. S. Ricietto, Carlos R. M. da Silva, Ivan R. Wolf, Gislayne T. Vilas-Bôas, Pedro M. O. J. Neves, Ana M. Meneguim, Laurival A. Vilas-Boas

**Affiliations:** a Departamento de Agronomia, Universidade Estadual de Londrina, Londrina, Paraná, Brazil; b Departamento de Biologia Geral, Universidade Estadual de Londrina, Londrina, Paraná, Brazil; c Laboratório de Entomologia, Instituto Agronômico do Paraná, Londrina, Paraná, Brazil

## Abstract

*Bacillus thuringiensis* is an important microbial control agent against insect pests. The draft genome sequence of the Brazilian strain BR58 described here contains the insecticidal genes *cry4A*, *cry4B*, *cry10A*, *cry11A*, *cry60A*, *cry60B*, and *cyt1A*, which show toxicity to both *Aedes aegypti* and *Hypothenemus hampei* larvae.

## GENOME ANNOUNCEMENT

The utilization of *Bacillus thuringiensis* as a natural biological control agent against agricultural pests is extensively known (1, 2). *B. thuringiensis* strains contain a property primarily related to the *cry*, *cyt*, and *vip* set of genes, which encode proteins active against insect pests. As previously reported, some *B. thuringiensis* strains are toxic toward two insect groups (3, 4). Nevertheless, strains with toxicity against *Diptera* and *Hypothenemus hampei* have been described only since 2003 (5). This insect is present in almost all coffee-producing countries (6), causing damages to crops and significant economic losses (7).

We present here the draft genome sequence of *B. thuringiensis* BR58, a Brazilian strain isolated from stored grains and with toxic activity against *Aedes aegypti* larvae (8), which also proved to be an efficient toxin-producing strain against *H. hampei* (9). The sequence was obtained using the Ion Torrent PGM system, which generated 2,312,798 reads, with an average length of 308 bp. We used an assembly strategy whereby we first mounted contigs for the chromosome and then assembled the plasmid contigs. For the first assembly, all reads were submitted to the Deaconess filtering (10). For this filtering process, we used the whole genome of all *B. thuringiensis* strains from the NCBI Genome Database (http://www.ncbi.nlm.nih.gov/genome/) available as of April 2015, and the 1,910,681 reads presenting similarity to the genomes already assembled were kept. The remainder, probably extrachromosomal content, was separated for use in plasmid assembly. The reads obtained after this filtering were assembled using the program MIRA version 4.0.2 (11), generating 138 contigs with an *N*_50_ of 207,956 and a total of 5,578,174-bp consensus sequences with a chromosome G+C content of 35%. The 402,117 remaining reads were used for plasmid construction, generating 123 contigs with an *N*_50_ of 17,777 and a G+C content of 34%.

These two groups of contigs were annotated separately using the RAST program SEED Viewer version 2.0. According to the RAST program, the contigs obtained from the chromosome showed 6,286 coding sequences and 173 RNA genes. Also, 482 subsystems were identified, and 85 coding sequences were involved in the virulence, disease, and defense subsystem. Regarding the plasmid sequences, 941 coding sequences and 16 RNA genes were annotated, 34 subsystems were identified, and only 7 coding sequences belonged to the virulence, disease, and defense subsystem category. We also found six *cry* genes, *cry4A*, *cry4B*, *cry10A*, *cry11A*, *cry60A*, and *cry60B*, and the *cyt1A* gene. Using these gene sequences and comparing them to all contigs using the Blast program, all the *cry* genes and the *cyt1A* gene were found in the plasmid contigs only. Full-length cloning experiments of the genes encoding insecticidal proteins carried by *B. thuringiensis* BR58 are essential to verify either the toxicity of each protein individually or the toxicity of combined proteins against *H. hampei* larvae.

### Nucleotide sequence accession numbers.

This whole-genome shotgun project has been deposited in GenBank under the accession numbers LIIT00000000. The version described in this paper is the first version, LIIT01000000.
